# Scrub Sponge as A Wound Debridement Device

**DOI:** 10.30476/BEAT.2021.85912

**Published:** 2021-04

**Authors:** Mehdi Ayaz

**Affiliations:** 1 Amiralmomenin Hospital, Shiraz Burn Research Center, Department of Surgery, Shiraz University of Medical Science, Shiraz, Iran

**Keywords:** Debridement, Sponge, Wound, Preparation, Burn

## Abstract

**Introduction:**

Wound debridement is necessary before skin grafting or wound closure. Inappropriate wound bed preparation will led to graft rejection and sometimes catastrophic results, especially in large wounds,. Usage of popular debridement and excision devices such as dermatomes has some difficulties and disadvantages. In this study we will introduce metallic scrub sponge as a safe and effective debridement device.

**Methods:**

The surgeon usually uses a sterile metallic scrub sponge over the wound with transverse or rotational repeated movement. Wound preparing with metallic sponge should be stopped when pinpoint bleeding occurs. We used sterile metallic sponge for more than 2500 burn patients.

**Results:**

The results are excellent for deep second degree burn (and deeper burns at least 5-10 days after burn when some eschar loosening occurs). Work with scrub sponge was effective, fast and safe.

**Discussion:**

Metallic scrub sponge is a useful device for wound preparation due to its some special characteristics. Debridement of the burn wound with metallic sponge can preserve the spontaneous epithelialization potential of skin in second degree burns and reduce additional injury to the viable tissue that is inevitable by surgical debridement. Cost effectiveness, easy accessibility, safety, softness, inertness and some others are among the other advantages of metallic sponge usage for wound preparation.

**Conclusion:**

Due to metallic sponge’s simplicity and capability to remove necrotic loose tissues and easy accessibility everywhere and minimal adverse effects, it is a good first line tool for wound preparation and debridement.

## Introduction

Wound preparation is necessary before wound closure or skin grafting. Skin graft will not take if recipient site is not properly prepared or ready for graft placement. It means wound bed should be viable, clean and free from any debris.

Many factors will affect graft take. At the first 48 hours of graft placement, graft nourishment is via imbibition. This nourishment will not be possible if there is any nonviable tissue on recipient bed or graft is not in immediate contact with viable recipient bed. For example, when there is sub-graft blood or clot, serum or air collection skin graft will fail. So, recipient site should be viable with immediate contact of skin with underlaying viable tissue after careful and enough debridement or excision. Remaining any dead tissues can lead to graft rejection and even infection or sepsis [1].

Wound preparation may need debridement or excision of eschar and necrotic tissues or only gentle scrubbing of wound with sterile gauze or tongue blade when necrotic tissues are absent as granulation tissue is formed. Excision or debridement is the usual and most popular and fast route for removal of dead tissues. Excision may be fascial or tangential. Fascial excision is more time consuming with less cosmetic results although bleeding in less than tangential excision. Tangential excision is most popular due to it’s fastness and better cosmetic results but bleeding is more in comparison to fascial excision (nearly 50-150cc blood loss per one percent excision for tangential excision related to day of excision). It requires sufficient experience though tangential excision is simple [2-4]. Tangential excision in the relatively inaccessible areas of body like face, axilla and perineum (concave areas) is very difficult [5].

As mentioned earlier, incomplete wound bed excision will be led to graft rejection especially in large wounds and catastrophic results. On the other hand, more viable tissue removal will at least lead to less cosmetic results (deeper wounds need to be covered with thicker skins with more dermis and resultant more donor site scar). 

Another adverse result of excessive dermal removal is penetration to sub-dermal fat with resultant less graft take (graft take is less successful if performed on sub-dermal fat than dermal tissue or fascia due to less blood supply of fat). Also, converting such tangential excision (that has exposed sub-dermal fat) to fascial excision to ignore fat as a non-ideal bed for grafting will leave an ugly healed wound.

Also, more excision of viable dermal tissue will make it thinner and resultant decreased skin pliability (pliability of skin mostly is related to dermis). So it is important to remove as less as possible viable tissues during excision.

Debridement or excision (tangential of fascial) of eschar tissue can be done with different instruments such as: metzenbaum scissor, scalpel, Goulian knife, Humby dermatome, electrical or powered dermatome, curette and versajet [6]. Mentioned devices have some advantages and disadvantages.

Some degrees of difficulties or need for more training, more time consumption, more blood loss, inadequate or excessive debridement are some of the disadvantages of debridement or excision with such devices. Perhaps we can say blood loss during operation and over-excision fear are among the most common causes of refusal for early excision and grafting in some centers around the world.

In short, usage of popular debridement and excision devices has some disadvantages:

- Danger of excision of more viable tissues and damage to tendons, nerves if used inappropriately in critical areas (such as back of hands, on bony prominencies and face) or used by untrained persons

- Possibility of damage to surrounding healthy tissues [7]

- Difficulty in debridement of softer areas such as the female breast and sunken or concave areas such as axilla

- Risk of diseases transmission between patient and surgeon or stuff due to inappropriate injury with blades [8]

- Need for training

- Expensiveness of devices and disposable blades

- Not available everywhere

Metallic scrub sponge, that is usually used in kitchen, is a useful device for this purpose (wound preparation or debridement) due to its some special characteristics. Usage of this simple device for debridement or complete removal of necrotic tissues is easy and fast. Debridement of the burn wound with metallic sponge can preserve the spontaneous epithelialization potential and reduce additional injury to the viable tissue that is inevitable by surgical debridement [9, 10].

**Fig. 1:**
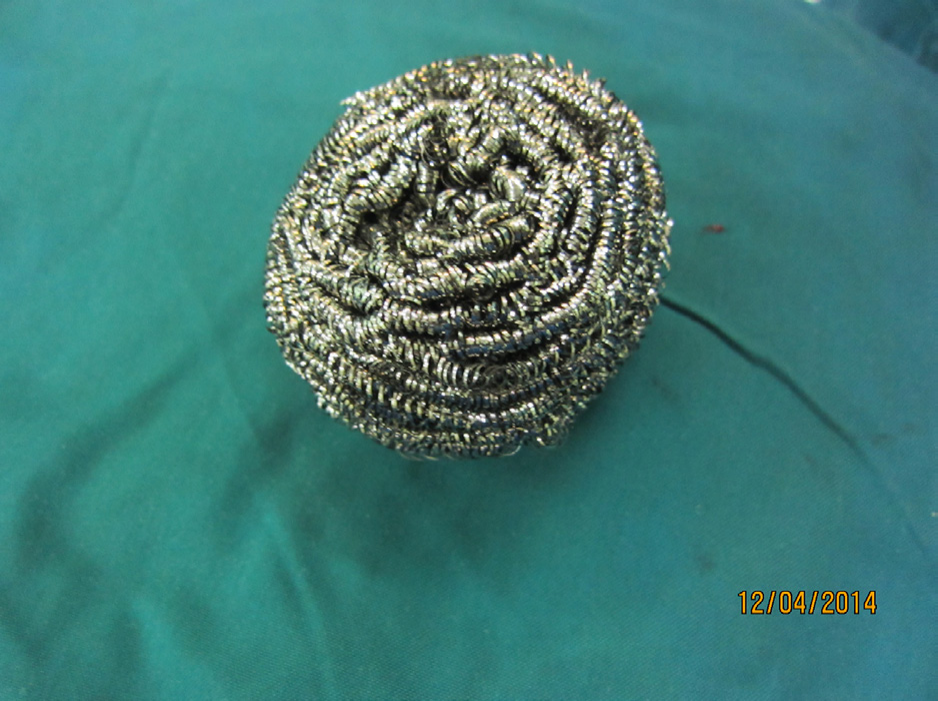
Scrub sponge that is used in kitchens for dishes cleansing

For these reasons, we recommend to use metallic scrub sterilized sponge for preparation of burn wound with some advantages and disadvantages (Figure 1).

Advantages of metallic sponge usage for wound preparation or debridement are:

- It is cheaper and most cost effective

- It is easily accessible (present in nearly all markets)

- Fast wound preparation (procedure duration is short)

- Less normal tissue damage below the wound such as tendons

- Usually only remove dead tissues, leave acceptable wound with pinpoint bleeding

- It does not harm surroundings normal epithelium even when used forcefully

- The device softness is not harmful for surgeon’s hand

- Easy to use and easy to train (inexperienced personnel even can use it)

- Preoperative blood loss is less (only due to bleeding from capillaries; sponge cannot injure larger vessels with resultant less bleeding)

- It is inert with no systematic or local reactions

- It is easy to sterile metallic sponge

- It is very useful for debridement of difficult areas such as interdigital webs, axilla, face, perineum, etc.

Disadvantages of metallic sponge usage for wound preparation are as follows:

- It can not remove thick eschar at early stages (although after several days due to some loosening of eschar it will be feasible)

- For preparing larger surface areas 2 or more of metallic sponges may be needed

- It should be used gently over granulation tissues because it can remove good vascularized granulation tissues and leave less vascularized fat or viable organs under it if used forcefully.

- Moving it over surrounding of recently healed skin can de-epithelialize it or very rarely if used very forcefully can superficially traumatize surrounding non-burnt skin.

In our burn center we use sterile metallic sponge for more than 2500 burn patients.

## Patients and Methods

**Fig. 2:**
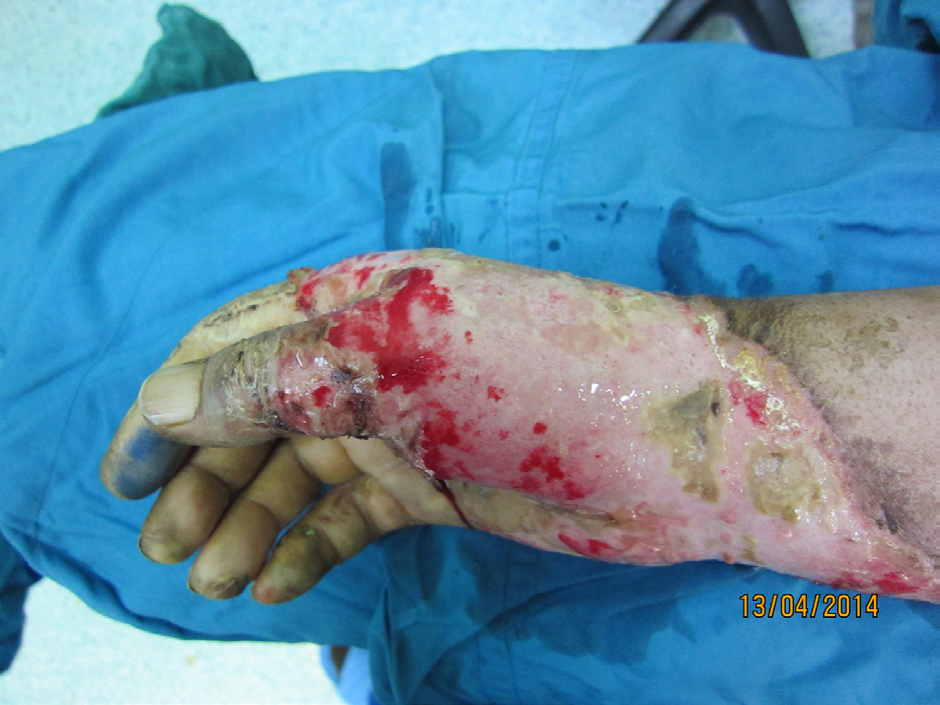
Deep second degree burn of right hand of a young man

**Fig. 3:**
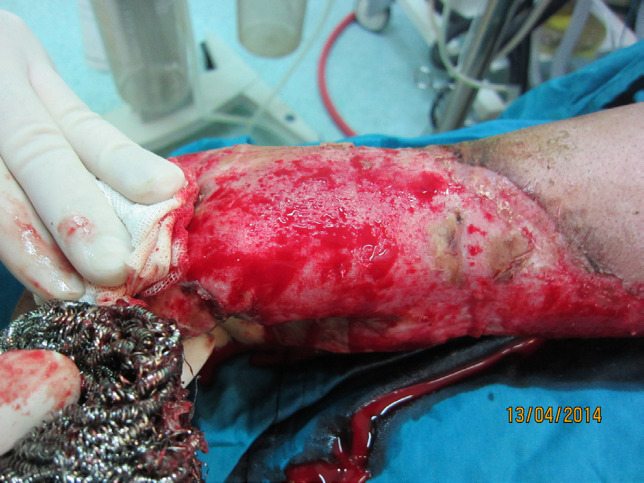
Debridement of the wound showed in figure 2 for skin graft

**Fig. 4:**
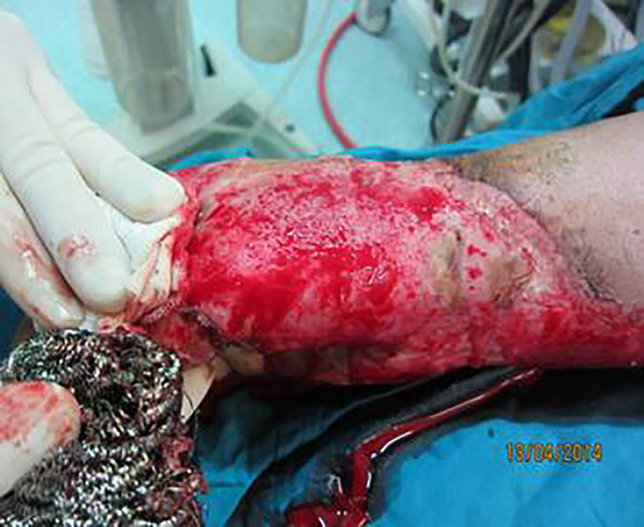
The Wound showed in figures 2 and 3 after debridement with sterile scrub sponge and ready for engraftment. See red wound bed with good pinpoint bleeding

**Fig. 5:**
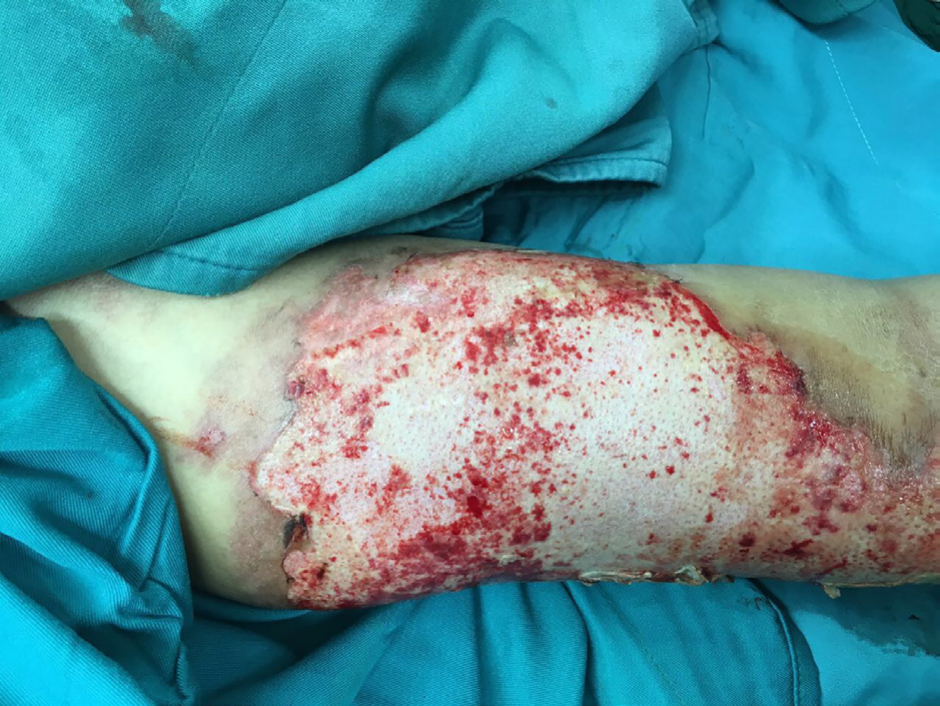
Deep second degree burn of trunk before debridement in the operating room

After preparation and drape of operation field and evaluation of wound and surrounding of intact or recently healed wound [Figures 2-5], the surgeon uses a sterile metallic scrub sponge [Figure 3]. The surgeon usually uses sponge over the wound with transverse or rotational repeated movement with low pressure at first and then continues while increasing the force. Although movement of sponge with forceful pressure on unburnt skin of surrounding of the wound usually is not harmful and don’t cause abrasion on intact skin, but forceful movement of sponge over recently healed area around the wound will cause de-epithelialization (a disadvantage of this device). Otherwise, usage of metallic sponge with very forceful pressure on intact non-burnt skin can cause skin abrasion. All of these abrasions will heal less than one week.

We recommend to apply medium force to prevent inadvertent damage of surrounding normal intact or recently healed fragile epithelium. Surgeon should exam the wound intermittently after cleaning oozed blood. The procedure usually is repeated several times at a session to decide if the wound is ready for graft placement or not. The surgeon can repeat the procedure again. In each field, the procedure takes a few seconds. Metallic sponge wound preparing should be stopped when pinpoint bleeding occurs or there was no progress in wound debridement. If metallic sponge couldn’t remove more eschar or debris, then the surgeon should use a sharp debridement device such as Goulian knife to compete the debridement

Metallic sponge is not useful for third degree burns in the first 10-14 days after burn injury due to eschar elasticity and rigidity.

After 14-10 days eschar is softer in third degree burns and can be removed more easily by sponge, especially from periphery (eschar loosening usually begins from periphery of wound). For deep 2nd degree burn the result is better 5-10 days after burn. After trying with metallic sponge, the surgeon should re-evaluate the wound, and if needed he/she can repeat the procedure again. As eschar loosens from periphery it will be easier to perform debridement of this area of wound at earlier time. In many wounds it may not be possible to remove all of the necrotic tissues with metallic sponge and after some trying with metallic sponge we should remove hard eschars with other devices such as Goulian. However we recommend metallic sponge as a prerequisite or auxiliary device for debridement or wound preparation.

## Results

We used sterile metallic sponge for more than 2500 burn patients. The results were excellent for deep second degree burn (and deeper burns at least 5-10 days after burn when some eschar loosening occurs). Work with scrub sponge was effective, fast and safe.

## Discussion

Surgically, removal of eschar tissue is the most important part of treatment of deep second degree burn and deeper. This part of surgical management of burnt wound could be performed by many instruments and in different ways. However careful dead tissues removal is very important part of this surgical procedure as less or more eschar removal can complicate the treatment result.

Skin graft will take only if the wound bed is properly prepared and it’s viability is related to wound bed viability. Any necrotic tissues will compromise graft take and it’s viability. 

Therefore removal of all of the necrotic tissues (eschar) is very important before graft placement.

There are many devices for this purpose, the most popular devices are Goulin knife and Humby dermatome [6]. These devices have some advantages and disadvantages. Danger of inadvertent operator injury is probable due to sharp edges of nearly all of these popular debridement or excision devices.

On the other hand, they could damage underlying and surrounding viable tissues especially tendons and joints, if handled carelessly [7]. 

Therefore training is very important for surgeons or operators to avoid these problems. As indicated less excision could end to graft failure and more excision of viable tissues will make the wound larger and deeper with resultant more difficult management. Otherwise, dermatomes especially the electrical ones and their disposable blades are expensive.

Another problem occurs when we are going to excise or performing debridement of inaccessible areas like face, interdigital webs, axilla, popliteal or perineum areas [5]. Debridement and excision of such areas is very difficult with devices that use blades. Due to the concavity of these areas debridement with straight long instruments is very difficult. Another problem with these devices is noticeable blood loss that may be due to more removal of underlying or surrounding viable tissues and cutting larger vessels. 

Difficulty in debridement of softer areas such as the female breast is another problem when using these instruments. The last problem is the risk of disease transmission due to inadvertent injury.

**Fig. 6:**
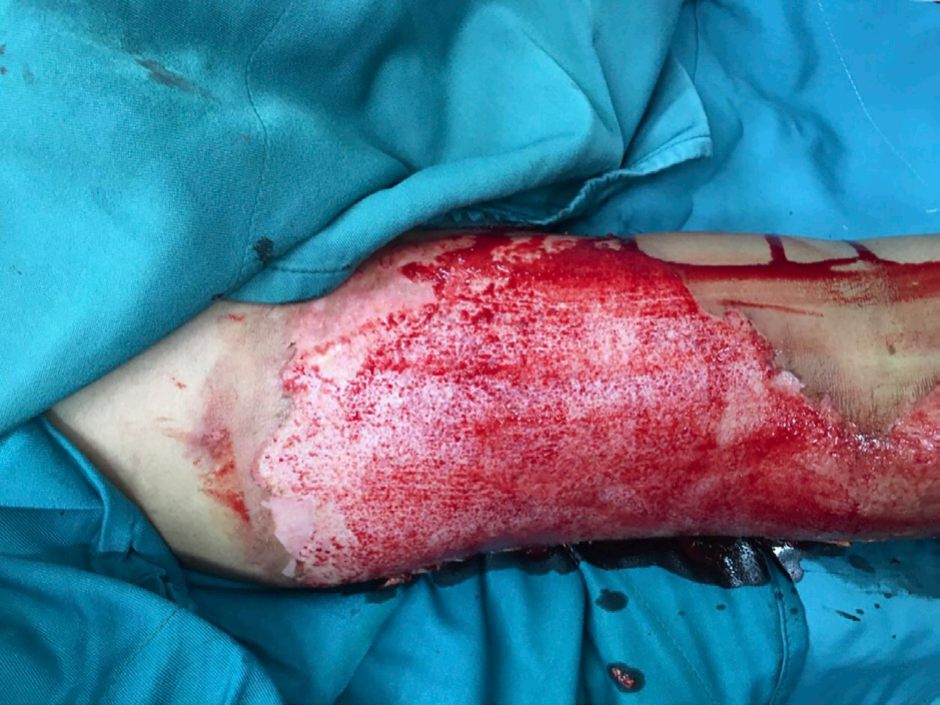
The wound showed in figure 5 after debridement with sterile scrub sponge and ready for engraftment. See red wound bed with pinpoint bleeding

Therefore, we are introducing a familiar device for wound debridement. Metallic scrub sponge [Figure 1], that is usually used in kitchen, is a useful device for wound debridement and preparation for skin graft due to its some special characteristics. Usage of this simple device for debridement or complete removal of necrotic tissues is easy and fast [Figures 4-6]. Burn wound debridement with metallic sponge can preserve the spontaneous epithelialization potential and reduce additional injury to the viable tissues that is inevitable by surgical debridement [9, 10].

Scrub sponges are cheap and we can buy one or more of metallic sponges with less than 1$. Metallic sponges are easily accessible and we can buy them in any market. It’s usage is easy as every person can use it correctly at the first time in the kitchen for cleaning dishes; the technique is usually the same for both dish washing and wound preparation with some little differences. It is soft and not harmful for surgeon’s hands. 

No specific cautions are usually necessary due to its simplicity and safety for operator’s hands and patient’s normal intact skin and the procedure will be safe. As it was mentioned before and due to its soft spongy consistency, metallic sponge can not damage intact tissues and surrounding skin when used with moderate force.

If used carefully, metallic sponges will remove necrotic tissues and fibrins due to their soft and spongy consistency. 

Minimal capillary bleeding usually responds to topical warm adrenalin/saline (1/10,000). 

Therefore this device usually can not rupture larger vessels so blood oozing is easily controllable with packing. Metallic sponge is inert with no reactions to body. 

It’s sterilization is easy as it is a metal. Soft consistency of sponges has made these devices as a very useful instruments for debridement and preparing difficult and concave areas too.

For these reasons, we recommend usage of sterile metallic scrub sponge for preparation of burn wound for skin graft. This is applicable for other wounds too.

In spite of many advantages of metallic sponges for wound preparation, we found some precautions and limitations when using it for debridement. It can not remove thick eschar although after several days due to some loosening of eschar it will be feasible. Also, it should be used gently over granulation tissues because it can remove good vascularized granulation tissues and leave less vascularized fat or viable organs under it. Moving metallic sponges over surrounding of recently healed skin can de-epithelialize it or very rarely if used very forcefully can superficially traumatize surrounding non-burnt skin. However de-epithelialization will heal in less than a week. For preparing larger surface areas 2 or more of metallic sponges may be needed or we should wash and re-use them.

## Conclusion

Metallic sponge, a simple tool that is usually used for dish cleaning in kitchen, has many capabilities that made it as a good wound preparing instrument. We used it after sterilization for wound preparation before grafting. Due to it’s simplicity and capability to remove necrotic loose tissues and easy accessibility everywhere and minimal adverse effects, it is a good first-line tool for wound preparation.

We recommend usage of metallic sponge before every debridement or excision and then completing the procedure with some kind of dermatomes or other sharp debridements if wound preparation with metallic sponge is not sufficient or successful.

## Ethics approval:

Not applicable.

## Funding:

Not applicable.
